# Dolutegravir Is Associated With Lower Odds of Gestational Diabetes Compared to Other Antiretroviral Therapy Regimens Among Pregnant Women in India: A Cross‐Sectional Analysis

**DOI:** 10.1002/jia2.70200

**Published:** 2026-09-20

**Authors:** Puja Chebrolu, Farheen Ahmed, Sanjaykumar Tambe, Mallika Alexander, Priyanka Raichur, Aditi Kole, Sonali Kante, Vandana Kulkarni, Nikhil Gupte, Ramesh Bhosale, Shilpa Naik, Amita Gupta, Todd T. Brown, Jyoti S. Mathad

**Affiliations:** ^1^ Weill Cornell Medicine New York New York USA; ^2^ University of Pittsburgh Pittsburgh Pennsylvania USA; ^3^ Byramjee Jeejeebhoy Medical College Pune India; ^4^ Byramjee Jeejeebhoy Government Medical College‐Johns Hopkins University Clinical Research Site and Center for Infectious Diseases in India (CIDI) Pune India; ^5^ Byramjee Jeejeebhoy Medical College Clinical Trials Unit Pune India; ^6^ Johns Hopkins University School of Medicine Baltimore Maryland USA

**Keywords:** antiretroviral therapy, dolutegravir, gestational diabetes, pregnancy, protease inhibitors, weight gain

## Abstract

**Introduction:**

In non‐pregnant adults living with HIV, dolutegravir is associated with weight gain and increased type 2 diabetes risk. In pregnancy, data on the effect of dolutegravir on gestational diabetes (GDM) risk is inconclusive. Data are particularly limited in Asian populations, where diabetes risk is especially high and not always associated with weight gain. We examined the association of dolutegravir with GDM among women living with HIV in India.

**Methods:**

We combined data from two cohorts of pregnant women living with HIV conducted at BJ Government Medical College in Pune, India—the PRACHITi study (2016−2019) and the PRAGATHi study (2023−present). Women were screened for GDM with the fasting 2‐h 75 g oral glucose tolerance test and diagnosed by World Health Organization criteria. Demographics, anthropometrics, antiretroviral therapy (ART), CD4 count and HIV viral load were collected at screening. Multivariate logistic regression was used to determine the association between current dolutegravir use and GDM.

**Results:**

Of 254 women living with HIV, 99.2% (*n* = 252) were on ART, of whom 66.3% (*n* = 167) were on dolutegravir‐, 22.6% (*n* = 57) were on efavirenz‐ and 6.3% (*n* = 16) were on protease inhibitor (PI)‐based ART. Overall, median CD4 count was 549.5 cells/mm^3^ (IQR 401−753), and 82.2% (*n* = 198) had undetectable viral load. Median body‐mass index (BMI) was 21.3 kg/m^2^ (IQR 19.4−23.8). The prevalence of GDM was 11.8% (*N* = 30). Among women on dolutegravir‐based ART, the prevalence was 10.8% (*N* = 18); among women on efavirenz‐based ART, the prevalence was 12.3% (*N* = 7), and the prevalence was 25% (*N* = 4) among women on PI‐based ART. Compared to women not taking dolutegravir, women taking dolutegravir had similar age and BMI but greater weight gain from the second to the third trimester (3.7 vs. 2.9 kg, *p* <0.01) and infant birthweight percentile (14.2 vs. 6.9, *p* <0.01). On multivariate logistic regression, dolutegravir use was associated with lower odds of GDM than other ART (aOR 0.2, 95% CI 0.1−1.0) irrespective of viral load and time on ART. Older age (aOR 1.1, 95% CI 1.1−1.2) and second‐trimester BMI (aOR 1.2 per kg/m^2^, 95% CI 1.1−1.3) were also associated with GDM.

**Conclusions:**

Women on dolutegravir had lower odds of GDM compared to women taking other ART, despite greater weight gain. Ongoing studies are investigating the possible anti‐inflammatory mechanisms.

## 1 Introduction

The World Health Organization recommends dolutegravir‐based antiretroviral therapy (ART) as first‐line therapy for people living with HIV, including pregnant women [1]. This recommendation is based on data showing that dolutegravir‐based ART resulted in a more rapid decline in viral load compared to other regimens [2]. In non‐pregnant adults, dolutegravir is associated with weight gain and increased risk of type 2 diabetes through adipose tissue fibrosis, insulin resistance related to lipid accumulation and increased adipogenesis [3, 4]. In pregnancy, data on the effects of dolutegravir and other integrase strand transfer inhibitors on metabolic disease, such as gestational diabetes (GDM), are limited.

GDM is a metabolic disorder of pregnancy that is linked to adverse maternal and neonatal outcomes, including preeclampsia, macrosomia, and future type 2 diabetes (T2DM) in the mother and child [5]. Despite the established link between dolutegravir and type 2 diabetes in non‐pregnant populations, there is limited data on the effect of dolutegravir on GDM. A study in Botswana found that women on dolutegravir had a 60% lower risk of developing GDM compared to those on efavirenz‐based ART. This cohort had a high median BMI of 26.1 kg/m^2^ [6]. But in a multi‐centre randomized controlled trial including 643 women taking dolutegravir‐ or efavirenz‐based ART in Botswana, Brazil, Uganda, Zimbabwe, Tanzania, South Africa, USA, Thailand and India, there was no difference in fasting glucose or haemoglobin A1c during pregnancy between arms. Notably, <3% of people in this study were from Asia [7].

In Asia, many women with diabetes are not overweight [8]. In normal‐ or low‐weight Asian people, diabetes is more related to insulin insufficiency rather than resistance [9]. Our studies and others have shown that Asian women with GDM are also more likely to have normal/low birthweight babies (<2.5 kg), as opposed to large babies (>3.5 or 4 kg) [10]. Given that dolutegravir is known to cause weight gain and increase adipose tissue insulin resistance, it is unclear if dolutegravir is helpful in Asian populations (due to increased weight gain and associated higher birthweight), or harmful (due to increased insulin resistance with underlying insulin deficiency). Understanding the effect of dolutegravir on GDM is important in improving our understanding of the pathophysiology and appropriate management of GDM. Our objective was to determine the effect of dolutegravir on GDM in a cohort of women living with HIV in India.

## 2 Methods

### 2.1 Study Design and Population

We included data from two prospective longitudinal cohorts of pregnant women living with HIV conducted at BJ Government Medical College, Pune, India—the PRACHITi study (2016–2019) and the ongoing PRAGATHi study (2023–present).

Inclusion and exclusion criteria for the PRACHITi cohort have been published previously [11]. Both studies recruited women >18 years of age in their second or third trimester (>20 weeks gestation) and excluded women with haemoglobin <7.5 mg/dL, weight ≤ 35 kg (≤ 40 kg in PRAGATHi), pre‐pregnancy diabetes, tuberculosis in the past 2 years or a history of autoimmune disease or immunosuppression besides HIV and diabetes. Women were enrolled within 1 month of screening. Dolutegravir + tenofovir disoproxil fumarate + lamivudine became first‐line ART in India in 2020.

### 2.2 Study Outcomes and Predictors

The primary outcome was GDM, defined according to the World Health Organization criteria using a universal fasting 75 g oral glucose tolerance test (OGTT) (any one abnormal value constituted GDM: fasting glucose >91 mg/dL, 1 h glucose >179 mg/dL or 2 h glucose >152 mg/dL) [12]. Women fasted for at least 8 h prior to OGTT, which was administered for research purposes. Current and prior ART regimens were recorded at screening. In cases where women were on both dolutegravir and a protease inhibitor (PI), they were included in the PI group. Other data collected included age, pregnancy weight gain, BMI, mid‐upper arm circumference (MUAC), ART regimens, duration of ART, CD4 count and HIV viral load at enrolment [13, 14]. Anthropometrics and blood pressure were measured using WHO STEPS guidelines [15].

### 2.3 Statistical Analysis

Statistical analyses were performed using STATA version 15 (Cary, NC). Descriptive statistics were used to compare women with and without dolutegravir exposure. Bivariate and multivariate logistic regression models were used to estimate associations between ART use and odds of GDM. Variables included in the multivariate model were determined based on known GDM risk factors. A sensitivity analysis limited to women on either dolutegravir or efavirenz was conducted using multivariate logistic regression.

### 2.4 Ethical Considerations

Studies received approval from the Byramjee Jeejeebhoy Government Medical College, Weill Cornell Medicine, and Johns Hopkins University IRB/IECs and adhered to ethical guidelines. Written informed consent in Hindi, Marathi or English was obtained from all participants prior to enrolment.

## 3 Results

A total of 254 pregnant women living with HIV were included in the combined cohort. Basic demographics were similar between cohorts (data not shown). Median age was 25 years (IQR 22−29), and median gestational age at entry was 20.2 weeks (IQR 16.6−24.6). Of the 99.2% (*n* = 252) on ART, 65.7% (*n* = 167) were on dolutegravir‐based ART, 22.6% (*n* = 57) were using efavirenz‐based ART, 16 (6.3%) were using PI‐based ART and 4.3% (*n* = 11) were using nevirapine‐based ART (Table 1). The median time on any ART for all women was 4.7 years (IQR 0.7–8.2). Among women using dolutegravir‐based ART, median time on dolutegravir was 2.2 years (IQR 0.4–3.2), and 31.1% (*n* = 52) had only been on this regimen. The most common regimen prior to dolutegravir was efavirenz (*N* = 57, 22.4%). The median CD4 count among all women was 549.5 cells/mm^3^ (IQR 401–753), with 82.2% (*n* = 198) having an undetectable viral load. Of those with a detectable viral load, the median viral load was 302 copies/mL (IQR 119−5089).

**TABLE 1:** Characteristics of women on dolutegravir versus not on dolutegravir.

	Total median (IQR) or *N* (%) *N* = 254	Dolutegravir *N* = 167	No dolutegravir *N* = 87
Age, years	25 (22−29)	25 (22−29)	25 (21−30)
CD4 count, cells/mm^3^	549.5 (401−753)	591.5 (424−772)	471.5 (342−632)
Undetectable viral load (<40 copies/mL)	198 (82.2%)	139 (90.3%)	59 (67.8%)
Viral load among detectable (copies/mL)	302 (119−5089)	116 (64−500)	718.5 (159.5−9604.5)
Current ART			
Dolutegravir^a^	167 (65.8%)	167 (100%)	NA
NNRTI‐based therapy	68 (26.8%)	NA	69 (75%)
‐Efavirenz	57 (22.4%)	NA	57 (62.0%)
‐Nevirapine	11 (4.3%)	NA	12 (13.0%)
Protease inhibitor‐based therapy	16 (6.3%)	NA	16 (17.4%)
No therapy	2 (0.8%)	NA	2 (2.2%)
Unknown type	1 (0.4%)	0	1 (1.1%)
Dolutegravir as first ART	52 (20.5%)	52 (31.1%)	NA
Time on ART, years	4.7 (0.7−8.2)	5.9 (2.3−9.4)	1.8 (0.1−5.3)
Time on dolutegravir, years		2.2 (0.4−3.2)	NA
BMI, kg/m^2^	21.3 (19.4−23.8)	21.2 (19.4−23.7)	21.4 (19.3−24.3)
High blood pressure (>130/80 mm/Hg)	12 (4.7%)	8 (4.8%)	4 (4.6%)

Abbreviations: ART, antiretroviral therapy; BMI, body mass index; IQR, interquartile range; NNRTI, non‐nucleotide reverse transcriptase inhibitor.

^a^ *N* = 160 participants were taking tenofovir+lamivudine+dolutegravir and *N* = 2 participants were taking zidovudine+lamivudine+dolutegravir.

Compared to women on non‐dolutegravir‐based ART, those on dolutegravir‐based ART had similar age (25 vs. 25 years, *p* = 0.26), BMI (21.2 vs. 21.4 kg/m^2^, *p* = 0.86), MUAC (23.8 vs. 23.5 cm, *p* = 0.47) and prevalence of high blood pressure (4.8 vs. 4.6% *p* = 0.95). Women using dolutegravir‐based ART had a significantly higher median CD4 count (591.5 vs. 471.5 cells/mm^3^, *p*<0.01) and undetectable viral load (90.3% vs. 67.8%, *p*<0.01). Women using dolutegravir also had greater weight gain from the second to third trimester (3.7 vs. 2.9 kg, *p*<0.01) and had infants with higher birthweight percentile (13.6 vs. 7.3, *p*<0.01) compared to women not on dolutegravir. The most common regimen prior to dolutegravir was efavirenz‐based (Table 2).

**TABLE 2:** Antiretroviral therapy regimens prior to switching to dolutegravir.

	Total *N* = 162	GDM *N* = 17	No GDM *N* = 145
Previous ART			
None	52 (32.1%)	4 (23.5%)	48 (33.1%)
Efavirenz‐based	57 (35.2%)	7 (41.2%)	50 (34.5%)
Nevirapine‐based	16 (9.9%)	3 (17.7%)	13 (9.0%)
Protease inhibitor‐based	10 (6.2%)	1 (5.9%)	9 (6.2%)
Unknown	27 (16.7%)	2 (11.8%)	25 (17.2%)

Abbreviations: ART, antiretroviral therapy; GDM, gestational diabetes.

The overall prevalence of GDM was 11.8% (*N* = 30). GDM prevalence was 10.8% (*N* = 18/167) among women taking dolutegravir versus 13.8% (*N* = 12/87) among women not taking dolutegravir (*N* = 7/57, 12.3% on efavirenz‐based ART). There was no difference in CD4 count or viral load between women with and without GDM. On multivariate logistic regression analysis, dolutegravir use was associated with lower odds of GDM (adjusted odds ratio [aOR] 0.23, 95% CI 0.06–1.01) independent of virological suppression, time on ART, age (aOR 1.15, 95% CI 1.05–1.25) and second‐trimester BMI (aOR 1.19, 95% CI 1.08–1.30) (Table 3). On comparison of women on dolutegravir with women on efavirenz, odds of GDM among women on dolutegravir were 0.39 (95% CI 0.11, 1.34), independent of virologic suppression, time on ART, age and second‐trimester BMI (data not shown).

**TABLE 3:** Univariate and multivariate logistic regression of the odds of gestational diabetes.

	GDM *N* = 30	No GDM *N* = 224	OR (95% CI)	aOR (95% CI)
ART				
Dolutegravir	18 (10.8%)	149 (89.2%)	0.4 (0.1, 1.2)	0.2 (0.1, 1.0)
NNRTI	8 (11.8%)	60 (88.2%)	0.4 (0.1, 1.5)	0.6 (0.1, 2.6)
PI	4 (25.0%)	12 (75.0%)	Ref	Ref
Age, years	28 (25.5−31)	24 (21−27)	1.1 (1.1, 1.2)	1.1 (1.1, 1.3)
BMI, kg/m^2^	25.0 (22.1−29.8)	21.1 (18.9−23.8)	1.2 (1.1, 1.3)	1.2 (1.1, 1.3)
MUAC^a^, cm	26.5 (23.3−30.1)	23.5 (22−26)	1.2 (1.1, 1.3)	
Weight gain from entry to third trimester	3.1 (1.7, 6.7)	3.6 (2, 6)	1.0 (0.9, 1.0)	
High blood pressure (>130/80 mmHg)	4 (33.3%)	8 (66.7%)	4.0 (1.2, 13.4)	
CD4 count (>350 cells/mm^3^)	25 (12.6%)	173 (87.4%)	1.4 (0.5, 4.0)	
Undetectable viral load (≤40 copies/mL)	28 (14.1%)	170 (85.9%)	3.4 (0.8, 14.8)	4.9 (0.9, 26.1)
Time on ART, years	5.0 (1.8, 10.9)	4.7 (0.4, 8.0)	1.0 (1.0, 1.1)	1.1 (1.0, 1.2)

Abbreviations: ART, antiretroviral therapy; BMI, second‐trimester body mass index; MUAC, mid‐upper arm circumference; NNRTI, non‐nucleotide reverse transcriptase inhibitor; PI, protease inhibitor.

^a^ Not included in multivariate analysis due to collinearity with BMI.

## 4 Discussion

We found that women taking dolutegravir‐based ART had a lower likelihood of developing GDM compared to women on non‐dolutegravir ART regimens, independent of age and pregnancy BMI. Despite the lower odds of GDM, women on dolutegravir had greater weight gain during pregnancy. Understanding the mechanisms of how ART affects GDM pathophysiology is important for prevention and treatment of GDM in people living with HIV, but also for the evaluation of future ART regimens, including long‐acting ART.

Women living with HIV who were on regimens other than dolutegravir‐based ART had higher odds of GDM compared to women taking dolutegravir‐based ART. The higher odds of GDM were primarily driven by women taking PIs. In non‐pregnant women, PIs are associated with increased risk of diabetes due to peripheral lipodystrophy and altered insulin signalling, which leads to insulin resistance. Pregnant women on PI therapy may, therefore, have higher baseline insulin resistance, increasing the risk of GDM. Interestingly, on comparison of dolutegravir‐ to efavirenz‐based therapy, we saw a trend towards greater odds of GDM in the women on efavirenz. Efavirenz has not been associated with diabetes in non‐pregnant populations. However, it has less potent anti‐inflammatory and antiviral effects compared to dolutegravir. Persistent inflammation may play a role in GDM risk because it leads to impaired glucose receptor action [16]. In our placental studies in the PRACHITi cohort, we found that women living with HIV had greater CD8 T cell infiltration compared to women without HIV [17]. In line with our current findings, a study of 323 pregnant women in Botswana with a median BMI of 26 kg/m^2^, 61% of whom were on dolutegravir and 39% on efavirenz, found that dolutegravir was associated with an OR of 0.40 (95% CI 0.18, 0.92) for GDM compared to efavirenz [6]. However, in the IMPAACT2001/VESTED trial, a randomized controlled trial comparing initiation of dolutegravir‐based versus efavirenz‐based ART in pregnancy, there was no difference in GDM prevalence between groups [7, 18]. Of note, VESTED was not powered to compare GDM prevalence and only included 18 Asian women, which may partially explain the difference in results.

Similar to non‐pregnant cohorts, we found that pregnant women taking dolutegravir had greater weight gain than women not taking dolutegravir. Interestingly, this weight gain did not increase the odds of GDM [3, 19]. Our cohort was predominately non‐overweight (median BMI 21.1 kg/m^2^), whereas studies showing an association of dolutegravir with type 2 diabetes in non‐pregnant, non‐Asian cohorts had higher baseline BMIs [20, 21]. Asian populations have a propensity towards insulin deficiency more than insulin resistance [22]. In studies comparing Asian adults with adults with GDM or T2DM in the United States and Europe, Asian adults consistently have up to three times lower insulin production than adults from other populations [9, 23]. In people with insulin‐deficient subtypes of diabetes, weight gain may actually be beneficial in augmenting insulin production by allowing beta cell hypertrophy, particularly during pregnancy [24−26]. Therefore, dolutegravir‐associated weight gain may be less metabolically adverse and actually helpful to increase birthweight in these women. Furthermore, dolutegravir also decreases inflammation, which decreases oxidative stress and pancreatic beta cell damage [27, 28]. Taken together, this suggests that dolutegravir could augment insulin production while decreasing insulin resistance. More studies are needed to understand this pathophysiology in both pregnant and non‐pregnant Asian women in order to guide prevention and treatment.

We also found that greater age and BMI were independent risk factors for GDM on multivariate analysis. This is in line with prior knowledge about GDM [5]. However, it is worth noting that the median pregnancy BMI among women who developed GDM was only 24.1 kg/m^2^ (IQR 21.6−30.1), and the median age was 30 years (IQR 28−34), both of which are below the thresholds that many guidelines suggest for determining if someone is high risk for GDM [29]. Our population is at high risk for GDM without classical risk factors, highlighting the importance of understanding the unique risk factors related to living with HIV and taking ART, especially in the setting of low BMI.

A key strength of this study is its use of data from two large longitudinal cohorts with robust follow‐up and high‐quality data collection. This allowed for adjustment for multiple potential confounders, including BMI, age and virologic suppression. The study was restricted to a single site in India, which may limit the generalizability of the findings to other ethnic and geographic populations. Similar studies should be done in countries where HIV and undernutrition are common. Future research should also focus on elucidating the mechanisms by which dolutegravir and other ART classes may influence glucose metabolism, including through insulin sensitivity, inflammatory cytokines and magnesium homeostasis, to help optimize prevention and treatment. Larger, multi‐centre studies can then examine the generalizability of these findings and their association with neonatal and long‐term postpartum outcomes.

Our findings provide novel insights into the differential metabolic effects of ART in pregnancy and suggest that dolutegravir‐based ART may have a lower risk of GDM than other ART. GDM should be considered an important clinical endpoint when examining new ART, including long‐acting regimens.

## 5 Conclusions

Indian women taking dolutegravir‐based ART had lower odds of GDM compared to women taking other ART. Mechanistic studies may help guide studies of future ART regimens.

## Author Contributions

PC and FA were involved with design, data analysis, writing and editing. ST, MA, PR, AK, PD, VK, NK, RB, SN, AG, TTB and JSM were involved with study design and manuscript editing. MA and JSM were additionally involved in study conceptualization and analysis. NG was involved in data analysis and manuscript editing.

## Funding

This work was supported by the National Institutes of Health Grant numbers K23AI129854, R01AI162235, R01HD081929, K23DK136388, K24AI120834, the Weill Cornell Medicine Fund for the Future Grant, and the Surgo Foundation.

## Conflicts of Interest

TTB has served as a consultant to ViiV Healthcare, Jannsen and EMD‐Serono.

## Data Availability

The data that support the findings of this study are available from the corresponding author upon reasonable request.
